# Royal Medical Benevolent College, Epsom

**Published:** 1903-06-27

**Authors:** 


### ROYAL MEDICAL BENEVOLENT COLLEGE, EPSOM.

### FESTIVAL BANQUET—SPEECHES BY THE PRINCE OF WALES AND LORD ROSEBERY.

MOST of the titled members of the medical profession, with a large number of the front rank and file—close on 450 guests in all—sat down at the 29th festival dinner of the Royal Medical Benevolent College, Epsom, held at the Hotel Cecil, on June 10th, under the presidency of the Prince of Wales.

The toast of "The King, the Patron of Epsom College," was proposed by the Prince, and accorded musical honours, after which Sir William Church proposed, in felicitous terms, "The health of the Queen, the Prince and Princess of Wales, and the other Members of the Royal Family," recalling the fact that the King, as Prince of Wales, was present, in 1855, when the college was opened by the Prince Consort, and again in 1895 to lay the foundation stone of the lower school. He also referred to the work done by the Princess Christian to improve hospital nursing and management. This toast, also, was enthusiastically received.

The Prince of Wales acknowledged the toast. He said: My Lords and Gentlemen,—On behalf of the Queen and of the Princess of Wales and the other members of my family, and on my own behalf, I beg to thank Sir William Church for the very kind and flattering terms which he has used in proposing this toast, and I am indeed most grateful to you all for the very hearty reception which you have given to it. On the Queen and the other members of my family I know that the medical profession may ever rely for hearty sympathy and ready support in their work, and in every undertaking which may tend to mitigate the sufferings of our fellow-creatures. (Cheers.) So far as the Royal Medical Benevolent College is concerned, I can assure you that the Queen remembers with great pleasure the visit she paid to the college with the King when he laid the foundation stone of the lower school eight years ago; and, as Sir William Church has reminded us, the Prince Consort opened the college almost 50 years ago; and on that occasion he was accompanied by the King. I believe that it was not very many years ago that the King took the chair here at one of your festival dinners, and on that occasion he got the largest collection of those days; but I hope that to-night we shall beat it. (Cheers.) I trust that my presence here this evening will be regarded as a proof that I continue to take the same interest in the college that my family have since its foundation. (Cheers.) As president of five of our large London hospitals, I know what splendid service is rendered by the medical profession to those institutions; and also, as president of the King's Hospital Fund for London, I fully realise the value of the gift of their experience and time in assisting those who control the administration of the Fund. This assistance is specially valuable in the all-important work of visiting and reporting upon the hospitals which are desirous of help from the Fund. Deeply impressed as I am with all we owe to the doctors and surgeons of this country, it gives me the greatest pleasure to take the chair here this evening; and I once more thank you all for the very hearty manner in which you have received this toast, and the way in which you have come here this evening to support me by your presence. (Cheers.)

Rising again to propose the toast of the evening, the Prince was greeted with another burst of applause. He said: It is now my pleasure to propose "Success to the Royal Medical Benevolent College." No excuse is necessary for pleading the cause on behalf of which we are assembled here to-night. The charitable institutions of our country are among those national characteristics of which we are justly proud (cheers); and I can hardly imagine that there are any more deserving of public support than those whose object is to help members of the medical profession or their families who through misfortune or otherwise are brought to reduced circumstances. I feel I am uttering a mere commonplace in stating that we all owe a deep debt of gratitude to the doctor. We cannot get on without him. He is with us on our entry into the world and when we leave it (laughter and cheers), and is our confidential friend from the cradle to the grave. We consult him, confide in him, trust him, though he often has to tell us disagreeable truths. There are few of us who have not reason to be deeply grateful for his knowledge, tender care, and patient watchfulness by which we have been brought through a severe and dangerous illness. Certainly I myself can speak feelingly as one of that number. (Cheers.) But I go further, for over and above our individual thanks you will, I know, agree that our medical profession has earned the heartfelt gratitude of the whole nation; for, thanks to the marvellous progress in medicine and surgery and individual knowledge and skill the life of our beloved Sovereign—(cheers)—was, under the blessing of Providence, preserved to us during the past year. We must also remember, in estimating the splendid work accomplished by our hospitals, that nine-tenths of the medical advice, and this is certainly the best in the country—(hear, hear)—is given practically gratuitously. But the debt to the doctor is not merely one which is to be considered as discharged as soon as his bill for professional service has been paid. (Hear, hear.) There remains the sense of gratitude, which, I am glad to say, often not only finds expression in a sincere and lasting friendship between doctor and patient, but in thank-offerings by which this institution, with other medical charities, has materially benefited. The question may be asked whether the medical profession should not be able to suppport its own charities without appealing to the public. In answer, I would remind you that the popular idea that doctors are rich men is a very erroneous one. (Hear, hear.) True it is that there are a few wealthy members of the profession; but these you may probably count on your fingers. I know also that great generosity is shown among these few to their necessitous brothers. Some are able to make a fair income, and perhaps to save money; but too many are underpaid and struggle in an overstocked market, and the fact remains that there is an enormous residuum of medical men who only live from hand to mouth. Another common idea seems to be that doctors live for ever. Unfortunately, here, again, is another fallacy. Too many succumb to a hard life of exposure, by which their constitutions are undermined, or to the dangers of infection. The two following cases are among those recently before the notice of the college. A medical man was summoned to go over the Wiltshire Downs to see a child who had been burned. On the way there he himself was struck by lightning and killed, leaving a widow and a large family. A surgeon-major in the Army Medical Staff was compelled to retire from the service on account of complete loss of sight resulting from overwork and hardship on active service. He has a wife and five young children, is not strong himself, possesses but limited means, and partly supports his mother. To meet this distress about £14,000 a year is given by three great medical societies in the metropolis—the Medical Benevolent Fund, of which my old friend Sir William Broadbent is president (hear, hear), the Society for the Relief of Widows and Orphans of Medical Men, and the Epsom Medical College. (Cheers.) The college was founded by Mr. John Propert in 1855 to supply the want which his experience as a family and medical practitioner forced upon his notice. The school supports 50 boys on the foundation, the boys being clothed, educated, and maintained. There are also 50 aged or necessitous medical men or their widows who receive £30 a year, and to maintain these £8,000 a year is necessary. I am very glad to have had the opportunity yesterday of personally visiting the College at Epsom. I was delighted with everything that I saw there; indeed, one could not help feeling that the wishes and aspirations of the founder have been to a great extent fulfilled. I heartily congratulate the headmaster, Mr. Hart Smith, on the happy and healthy-looking boys under his charge whom I had the pleasure of seeing in the cricket-field. (Cheers.) But I wish it to be clearly understood that this appeal is not for the school proper, which is conducted as a public school and is self-supporting; it is for necessary funds to support its medical foundation—that is, the education of the 50 foundationers, and to maintain the pensions to which I have just referred; and I am glad to be able to state that there are strong reasons for believing that if we receive to-night liberal support it will not be necessary to have recourse to further special appeals. My only regret is that this appeal has not been urged by someone more eloquent than myself—("No, no")—but none could speak with more profound conviction than I do of the worthiness of the claim which the Royal Medical Benevolent College has upon the sympathy and generosity of us all. (Cheers.) I now beg to propose, "Success to the Royal Medical Benevolent College," coupling with it the names of the president, my noble friend Lord Rosebery, and the treasurer, Dr. Holman. In its president the institution has to be congratulated; for Lord Rosebery, while ably fulfilling the duties of that office, proves himself to be a true friend and neighbour, showing a keen interest in the general welfare of the college as well as in the boys, their work and their recreation. (Cheers.) I feel it presumptuous for me to speak about Dr. Holman. His name is familiar to every one who knows Epsom College. He is the mainspring of its life. Associated with the charity since its foundation, he has for 16 years worked unceasingly as treasurer, and has left a lasting proof of his efforts as an honorary local secretary in the £8,000, which sum he has collected during 38 years in that office. I now ask you to drink the toast. (Loud cheers.)

The toast having been drunk, Lord Rosebery, as President of the College, responded. He said his task was happily a small one, being only to obliterate himself as quickly as possible in favour of his working partner Dr. Holman. (Laughter.) If he had had any intention of making an extended speech it would have been dispelled by that to which they had just listened. The only part of that speech with which he ventured to disagree was that they could have had a better Chairman than his Royal Highness. He dared challenge any orator, however practised, to vie with the practical, the eloquent, and the true terms in which his Royal Highness had spoken of the medical profession. There were so many members of it there that evening that he scarcely dared to conjecture what might be the effect on the people of London. (Laughter.) The titled cynics of the profession among whom he was sitting had given expression to opinions on the subject, but he would not venture to imitate them. He could only hope that all might be for the best. (Laughter.) He did not like to call himself an impostor, but strange as it might seem, he was an ornamental figure on that occasion. He was President of Epsom College, but he was responsible neither for its teaching nor its discipline. If he were he might have to give a less favourable report than that which he was then able to present. He would like to exchange offices with the Bishop of Winchester and become Visitor to the College. He was a neighbour at Epsom (cheers) and was frequently a visitor—much more frequently he suspected, owing to local circumstances, than any occupant of the See of Winchester. (Laughter.) He could offer no report on the studies; Mr. Hart Smith never asked his judgment on them. But regarding the athletic features of the College he could say from considerable experience—as his Royal Highness had been able to say from his experience of the previous day—that a finer and a manlier lot of students could exist in no establishment in the world. (Hear, hear.) They lived in the finest air; they were carefully kept from the temptations associated with the name of Epsom (laughter), and they were, he believed, prepared to go anywhere and do anything. (Cheers.) There was only one drawback connected with the College, and that he hoped might be removed as a result of that meeting—it was the word "Benevolent" in the title. (Hear, hear.) He had lived for years as President of the Royal Medical Benevolent College of Epsom; if he could die President of Epsom Medical College he would die content. He hoped it might be the task of his Royal Highness to accomplish that work. (Cheers.) There had been many Chairmen before him and many successful banquets, but he hoped when that was over they would be able to say that whereas Chairmen before him had collected their thousands he had collected his tens of thousands. (Cheers.)

The Secretary then read the list of subscriptions, which totalled £6,526, the announcement being received with applause.

Dr. Constantine Holman, the Treasurer, replying also to the toast, said that on behalf of his colleagues on the Council of the College he thanked his Royal Highness for his presence there and for the terms in which he had commended the cause of the pensioners and foundation scholars. That gathering was the largest ever seen in the metropolis in the cause of a medical charity, and he hoped his Royal Highness would accept it as the grateful recognition by the profession of his kindness in presiding. If John Propert, their founder, could see it he would be more than satisfied with the way his work had prospered. He should like to thank his Royal Highness very warmly for his speech. It contained much that had long been his own opinion on the relations between the public and the medical profession, and coming from his Royal Highness it would leave a stronger impression than coming from any medical man. His Royal Highness had touched upon the question of the earnings of medical men. Their work was personal, and their harvest time short, but he desired to bear testimony to the fact that they gave generously for the relief of the necessitous of their profession. It had been said that much good would accrue if ten shillings a year were contributed by each medical man, but if every practitioner in the United Kingdom would only give half-a-crown he would have no further anxiety for Epsom College. Dr. Holman said that to-night he was able to announce that the two daughters of the late Mr. Leach, of Shaw, near Oldham, had given the Council £1,000 to found a Leach Memorial Scholarship of £30 a year, tenable for four years, to be awarded without competition to a boy requiring assistance to enter either at Oxford or Cambridge, preference to be given to orphan sons of medical men. The Ainslie Memorial Scholarship, held for a like term, would now enable a scholarship to be awarded every two years. For 50 years they had worked hard and been honoured by the confidence of the Royal Family, and now, representing the third generation, his Royal Highness had come to their help. (Cheers.)

The Prince of Wales's departure was signalised by the company uprising and singing "For he's a Jolly Good Fellow," after which the proceedings terminated.

